# The predictive value of PCT, SP-D and 8-iso-PGF2 α for the development of severe pneumonia in children

**DOI:** 10.4314/ahs.v24i3.9

**Published:** 2024-09

**Authors:** Guiying Zhang, Yang Hu, Bo Huang, Hongliang Gao, Mengsha Zhu

**Affiliations:** Department of Critical Care Medicine, Hebei Children's Hospital, Shijiazhuang, China

**Keywords:** severe pneumonia in children, procalcitonin, 8-isoprostane F2α, pulmonary surfactant-associated protein D, disease assessment

## Abstract

**Background:**

To investigate the predictive value of predictive value of procalcitonin (PCT), 8-iso-prostaglandin F2α (8-iso-PGF2α) and pulmonary surfactant-associated protein D (SP-D) for the development of severe pneumonia in children.

**Methodology:**

Children with severe pneumonia were selected as the pneumonia group, and children with non-pulmonary respiratory diseases were selected as the control group. PCT, 8-iso-PGF2α, and SP-D were compared between the pneumonia group and the control group at admission; PCT, 8-iso-PGF2α, SP-D, and Acute Physiology and Chronic Health Evaluation (APACHE-II), Pediatric Critical Illness Score (PCIS) and Clinical Pulmonary Infection Score (CPIS) scores were compared between children with severe pneumonia with different pathogen infection types and different prognostic outcomes; and the correlation between APACHE-II, CPIS, and PCIS scores and PCT, 8-iso-PGF2α, and SP-D levels was analysed in children with severe pneumonia.

**Results:**

PCT, 8-iso-PGF2α and SP-D in pneumonia group were higher than those in control group. PCT, 8-iso-PGF2α, SP-D, APACHE-II, CPIS, and PCIS were lower in good prognosis children than in poor prognosis children. APACHE-II, CPIS, and PCIS scores were positively correlated with PCT, 8-iso-PGF2α, and SP-D levels in children with severe pneumonia (P < 0.05).

**Conclusion:**

PCT, SP-D and 8-iso-PGF2α levels relate to pathogen type, illness severity and prognosis in children with severe pneumonia. They can aiding early evaluation.

## Introduction

Severe pneumonia is a common respiratory emergency in children, causing continuous and recurrent damage to the respiratory system, which can easily lead to serious complications such as respiratory failure, cardiac arrhythmia and pulmonary heart disease, and is extremely dangerous^1^. At this stage, the clinical assessment of severe pneumonia in children is mainly based on physical examination combined with imaging, but this is limited by the lag between clinical signs and lung histopathological changes in children with severe pneumonia. In general, when signs, symptoms, or imaging features are present in a child, the disease has already progressed significantly^2^. Therefore, new methods need to be found to further assess the severity of severe pneumonia. Serological indicators are objective and easy to operate. Procalcitonin (PCT) is a commonly used biomarker of bacterial infection in clinical practice and has a high evaluation value for systemic serious infections^3^. 8-iso-prostaglandin F2α (8-iso-PGF2α) and pulmonary surfactant associated protein D (SP-D) are newly discovered cytokines in recent years, and studies^4^,^5^ have confirmed that 8-iso-PGF2α and SP-D are closely related to the occurrence and development of severe pneumonia . There are few clinical studies on the combined application of PCT, 8-iso-PGF2α, and SP-D in severe pneumonia in children, so its predictive value for severe pneumonia is unclear. In view of this, this study investigated the predictive value of PCT, 8-iso-PGF2α, and SP-D on the development of severe pneumonia in children for further evaluation, in order to provide some clinical reference.

## Patients and methods

### Patients

The clinical data of 198 children with severe pneumonia in our pediatric intensive care unit from January 2019 to December 2020 were retrospectively analysed and set as the pneumonia group (198 cases). And the clinical data of 90 children with non-pneumonic respiratory diseases in our hospital during the same period were also collected as the control group (90 cases).

**Inclusion criteria involved that:** 1. Pneumonia group was confirmed by etiological examination, in line with the diagnostic criteria for severe pneumonia in children 6; 2. All children underwent PCT, 8-iso-PGF2α, and SP-D on admission. The results were available and unambiguous; 3. Children's guardians were fully informed of the examination content.

**Exclusion criteria set as follows:** 1. Sepsis, viral hepatitis, peritonitis, pancreatitis and other infectious diseases that may lead to abnormal test results; 2. Congenital immunodeficiency disease; 3. Severe limb trauma or multiple organ failure; 4. Congenital mental retardation or severe mental illness, poor cooperation. All children had their guardians sign a consent form prior to enrolment. The study protocols were in accordance with the Helsingin Declaration.

## Methods

All children received biological marker tests at admission: 1. Serum PCT and 8-iso-PGF2α tests: 5 ml of fasting peripheral venous blood was collected, and the supernatant was taken after 10 min of differential centrifugation (3500 rpm, 12 cm). Serum 8-iso-PGF2α was detected by enzyme immunoassay, PCT was detected by immunoluminescence assay, and analysed by automatic biochemical analyzer (HITACHI, Japan, model 7600-030). 8-iso-PGF2α enzyme immunoassay kit was purchased from Ningbo Meikang Biotechnology Co., Ltd. (Ningbo, China). PCT immunoluminescence assay kit was purchased from Wuhan Saipei Biotechnology Co., Ltd. (Wuhan, China). All operations were performed according to the relevant reagent and instrument instructions; 2. SP-D examination of bronchoalveolar lavage fluid: hematuria and stool routine, coagulation function, blood pressure, cardiac function and other examination items were perfected before lavage. After consent and clear that the children had no contraindications, alveolar lavage was performed. After anesthesia and sedation, bronchoscopy was delivered into the trachea through the nose. Detailed exploration of tracheal and lobar bronchial morphology, mucosa, soft tissue conditions, 9% NaCl injection was injected through the working channel, and lavage fluid was aspirated. SP-D was detected by enzyme immunoassay and reagents were purchased from Ningbo Meikang Biotechnology Co., Ltd. (Ningbo, China). One day after admission, the children in pneumonia group were assessed using Acute Physiology and Chronic Health Evaluation (APACHE-II), Pediatric Critical Illness Score (PCIS) and Clinical Pulmonary Infection Score (CPIS). Among them, APACHE-II score was 71 points, CPIS score was 10 points and PCIS score was 110 points. APACHE-II, CPIS and PCIS scores were positively correlated with the severity of pneumonia.

### Observation indicates

(1). PCT, 8-iso-PGF2α, and SP-D expression levels were compared between the pneumonia group and the control group at admission.
(2). According to the etiological examination results of children with pneumonia, children with bacterial infection were divided into bacterial group, and children with viral, mycoplasma, and chlamydial infections were divided into non-bacterial group, and the PCT, 8-iso-PGF2α, SP-D expression levels and APACHE-II, CPIS, and PCIS scores of children with different types of pneumonia were compared.
(3). According to the prognosis of pneumonia group, the children who survived after systemic treatment and had no residual complications were divided into good prognosis group, and the children who died or had residual complications after ineffective treatment were divided into poor prognosis group. The PCT, 8-iso-PGF2α, SP-D levels and APACHE-II, CPIS, and PCIS scores of pneumonia children with different prognoses were compared.
(4). To analyse the correlation between APACHE-II, CPIS, PCIS scores at 1 day after admission and PCT, 8-iso-PGF2α, SP-D expression levels at admission in children with severe pneumonia.

### Statistical analysis

Statistical Product and Service Solutions (SPSS) 22.0 (IBM, Armonk, NY, USA) was used to analyse the data, t test was used for measurement data; x^2^ test was used for enumeration data; Pearson correlation was used to analyse the correlation between APACHE-II, CPIS, and PCIS scores and PCT, 8-iso-PGF2α, and SP-D levels in children with severe pneumonia; P < 0.05 was statistically significant.

## Results

### Comparison of general data between the two groups

There was no significant difference in age (t = 1.425), weight (t = 0.671), height (t = 1.078), and gender (x^2^ = 0.050) between the two groups (P > 0.05), as shown in Table 1.

**Table 1:** Comparison of general data between the two groups

Group	N	Age (years)	Body Weight (kg)	Height (cm)	Gender [n, (%)]	

					Male	Female
Pneumonia Group	198	5.53 ± 0.76	20.89 ± 1.56	114.35 ± 3.17	105 (53.03)	93 (46.97)
Control group	90	5.67 ± 0.80	21.02 ± 1.44	114.79 ± 3.30	49 (54.44)	41 (45.56)
t/x^2^		1.425	0.671	1.078	0.050	
P		0.155	0.503	0.282	0.824	

### Comparison of PCT, 8-iso-PGF2α and SP-D between the two groups

PCT (t = 67.179), 8-iso-PGF2α (t = 129.597), and SP-D (t = 110.519) were higher in the pneumonia group than in the control group (P < 0.05), as shown in Table 2.

**Table 2:** Comparison of PCT, 8-iso-PGF2α and SP-D between the two groups (x̅±s)

Group	N	PCT (ng/mL)	8-iso-PGF2α (pg/mL)	SP-D (mg/L)
Pneumonia Group	198	7.75 ± 0.89	35.53 ± 2.16	201.35 ± 10.23
Control group	90	1.26 ± 0.32	5.18 ± 0.76	74.43 ± 5.53
t		67.179	129.597	110.519
P		0.000	0.000	0.000

### Comparison of biological indicators and disease indicators of children with severe pneumonia of different infection types

Among 198 children with severe pneumonia, 115 cases of bacterial pneumonia and 83 cases of non-bacterial pneumonia were confirmed by etiological examination. PCT (t = 53.502), 8-iso-PGF2α (t = 15.522), SP-D (t = 14.385), APACHE-II (t = 22.644), CPIS (t = 25.260), and PCIS (t = 12.858) were higher in children with bacterial pneumonia than in those without bacterial pneumonia (P < 0.05), as shown in Table 3.

**Table 3:** Comparison of biological indicators and disease indicators between children with bacterial pneumonia and non-bacterial pneumonia (x̅±s)

Type of infection	N	Biological indicators			Disease related indicators (points)		

		PCT (ng/mL)	8-iso-PGF2α (pg/mL)	SP-D (mg/L)	APACHE-II	CPIS	PCIS
Bacterial group	115	16.52 ± 1.75	37.35 ± 2.58	215.40 ± 11.31	22.15 ± 1.33	8.79 ± 0.23	82.89 ± 2.15
Nonbacterial group	83	6.31 ± 0.68	32.43 ± 2.29	195.54 ± 9.79	18.53 ± 1.08	8.08 ± 0.20	79.35 ± 2.20
t		53.502	15.522	14.385	22.644	25.260	12.858
P		< 0.001	< 0.001	< 0.001	< 0.001	< 0.001	< 0.001

### Comparison of biological indicators and disease indicators in children with severe pneumonia of different prognosis

After treatment, 187 of 198 children with severe pneumonia had a good prognosis and 11 had a poor prognosis. Children with good prognosis had lower PCT (t = 82.586), 8-iso-PGF2α (t = 39.279), SP-D (t = 40.174), APACHE-II (t = 47.353), CPIS (t = 74.406), and PCIS (t = 51.562) than children with poor prognosis (P < 0.05), as shown in Table 4.

**Table 4:** Comparison of biological indicators and disease indicators in children with severe pneumonia of different prognoses (x̅±s)

Prognostic Outcome	N	Biological indicators			Disease related indicators (points)		

		PCT (ng/mL)	8-iso-PGF2α (pg/mL)	SP-D (mg/L)	APACHE-II	CPIS	PCIS
Good prognosis group	187	6.20 ± 0.52	31.37 ± 1.89	185.52 ± 7.38	18.15 ± 0.89	7.88 ± 0.24	72.43 ± 1.98
Poor prognosis group	11	18.52 ± 1.37	43.40 ± 2.61	245.53 ± 13.26	27.39 ± 1.75	9.37 ± 0.10	88.66 ± 2.67
t		82.586	39.279	40.174	47.353	74.406	51.562
P		< 0.001	< 0.001	< 0.001	< 0.001	< 0.001	< 0.001

### Correlation between PCT, 8-iso-PGF2α, SP-D Levels and disease indicators in children with severe pneumonia

Pearson analysis showed that APACHE-II score was positively correlated with PCT, 8-iso-PGF2α, and SP-D levels in children with severe pneumonia (r = 0.298, 0.376, 0.320, P < 0.05), CPIS score was positively correlated with PCT, 8-iso-PGF2α, and SP-D levels (r = 0.316, 0.422, 0.388, Fig. P < 0.05), PCIS score positively correlated with PCT, 8-iso-PGF2α, SP-D levels (r = 0.220, 0.312, 0.277, P < 0.05), as shown in Table 5.

**Table 5:** Correlation between PCT, 8-iso-PGF2α, SP-D levels and disease parameters in children with severe pneumonia

Indicators	APACHE-II		CPIS		PCIS	

	R	P	R	P	R	P
PCT	0.298	0.003	0.316	0.000	0.220	0.002
8-iso-PGF2α	0.376	0.001	0.422	0.001	0.312	0.000
SP-D	0.320	0.000	0.388	0.001	0.277	0.001

## Discussion

Severe pneumonia in children is characterized by strong harm, stubborn condition, difficulty in treatment, and easy protracted healing, and is a major disease endangering the health of infants. Infants under 6 years of age are the main affected group of this disease, which can cause sputum, shortness of breath, fever and other symptoms in children, and in severe cases, central respiratory failure, multiple organ dysfunction and even death^10^-^12^. Pulmonary inflammation caused by mycoplasma, viruses, bacteria and other infections is the main cause of severe pneumonia in children, and if not treated in time, it may lead to the spread of infection, increase the risk of complications such as pulmonary heart disease and septic shock, and endanger children's physical and mental health^13^. Clinical treatment of severe pneumonia in children is mainly based on the type of pathogens infected in children, the condition of targeted treatment, through antibacterial, cough, fever and other drug treatment can effectively alleviate the clinical symptoms of children and reduce the risk of disease progression^14^,^15^. Inaccurate disease assessment may delay the optimal timing of treatment in children, affect the treatment outcome, and increase the risk of disease progression. Therefore, early diagnosis and disease evaluation of severe pneumonia in children are of great significance and have a positive effect on optimizing treatment options, controlling the child's condition, reducing respiratory tract injury, and reducing the risk of complications. PCT, 8-iso-PGF2α, SP-D and other indicators have good objectivity and rapid acquisition of examination results, through the above biological indicators examination can provide reliable reference data for the treatment, disease evaluation and prediction of severe pneumonia in children.

Pathological changes usually exist before the clinical symptoms or signs of children with severe pneumonia are aggravated, and the etiological examination time is relatively long (usually 1-3 days). Therefore, it is difficult to accurately predict and evaluate the condition of early children by relying only on clinical manifestations, imaging results or etiological examination. Because the resistance of children is weak and the physical development is not mature, the failure to accurately evaluate the condition may interfere with clinicians to develop the optimal treatment plan and affect the overall treatment effect and physical development of children.

In this investigation, PCT, 8-iso-PGF2α and SP-D in children with severe pneumonia were higher than those in children with non-pneumonic respiratory diseases, which indicated that the combined detection of PCT, 8-iso-PGF2α and SP-D could preliminarily differentiate pneumonia from other non-pneumonic respiratory diseases in children after excluding the interference of other possible infectious diseases. The results also showed that the expression levels of PCT, 8-iso-PGF2α and SP-D were higher in children with bacterial severe pneumonia and poor prognosis severe pneumonia, and APACHE-II, CPIS and PCIS scores were positively correlated with PCT, 8-iso-PGF2α and SP-D levels in children with severe pneumonia, indicating that the combined detection of PCT, 8-iso-PGF2α and SP-D could predict and evaluate the infection type, disease condition and prognosis quality of severe pneumonia, thus providing a reference basis for the disease evaluation and clinical treatment of severe pneumonia.

This is due to the association between the occurrence and development of pneumonia and the level of oxidative stress in the body, which will promote the production of cellular reactive oxygen species and thus aggravate tissue injury and body inflammation, while 8-iso-PGF2α can increase vascular injury and lead to antioxidant – oxidative imbalance in the body, so its expression level will show an increasing trend with the aggravation of the condition of children with pneumonia. SP-D is an immunoregulatory protein that binds to pneumonia pathogens such as Mycoplasma pneumoniae and Gram-positive bacteria and regulates the clearance of pathogens by macrophages. Infection and invasion of pulmonary pathogens promote the secretion and expression of SP-D. Therefore, SP-D is usually highly expressed in patients with pulmonary infection^16^.

PCT is sensitive to bacterial infections and is not affected by inflammation of the body's metamorphic immune response, therefore expression levels in the serum of healthy individuals are extremely low and significantly elevated in the presence of lung infections, particularly bacterial infections^17^.

## Conclusion

In summary, the expression levels of PCT, SP-D and 8-iso-PGF2α are related to the type of infectious pathogens, severity of illness and prognosis quality of children with severe pneumonia. PCT, 8-iso-PGF2α and other indicators are easy to operate, rapid and easy to popularize, and can be used as predictors for early evaluation of the condition and prognosis of children with severe pneumonia.

## Data Availability

The datasets used and analysed during the current study are available from the corresponding author on reasonable request.
